# Supplementary material for: Do sugar-sweetened beverages cause adverse health outcomes in adults? A systematic review protocol
Source: Syst Rev. 2014 Sep 23;3:108. doi: 10.1186/2046-4053-3-108 (PMC4178316; doi:10.1186/2046-4053-3-108)
Supplement: Additional file 2 — Examples of validated generic quality of life instruments. This file provides examples of validated generic quality of life instruments. [file 2046-4053-3-108-S2.pdf]

Additional file 2

Information obtained from: PROQOLID – Patient-Reported Outcome and Quality of Life Instruments Database. http://www.proqolid.org

1. *15-Dimensional Health-Related Quality of Life Measure (15D)* - Assessment of health related quality of life and its utility in adults 16 years of age or over.
2. *Assessment of Quality of Life (AQoL)* – Assessment of health-related quality of life in adults with a multi-attribute utility measure.
3. *Centers for Disease Control and Prevention Health-Related Quality of Life Measure (CDC HRQOL-14)* – Assessment of health-related quality of life in adults and used to monitor population health levels in epidemiologic and prevention research.
4. *Duke Health Profile (DUKE)* – Assessment of health-related quality of life and functional health status in adults.
5. *Euroqol EQ-5D (EQ-5D)* – Assessment of health outcomes in adults and children 12 years of age or over.
6. *Ferrans and Powers Quality of Life Index (QLI)* – Assessment of quality of life in terms of satisfaction with life.
7. *Flanagan's Quality of Life Scale (QOLS)* – Assessment of quality of life in adults.
8. *Quality of Life Inventory (QOLI)* – Assessment of life satisfaction and outcomes based on 16 key areas of life in adolescents and adults.
9. *Quality of Life Questionnaire – Evans (QLQ-E)* – Assessment of quality of life in adults across a broad range of areas.
10. *Quality of Life Systemic Inventory (QLSI)* – Assessment of quality of life in adults.

11. *Quality of Well Being Scale (QWB)* – Assessment of health-related quality of life in adults.
12. *Schedule for the Evaluation of Individual Quality of Life (SEIQoL)* – Assessment of quality of life in adults.
13. *Short Form-12 Health Survey (SF-12)* – Shorter alternative to the SF-36 for use in adolescents (14 years of age or over) and adults.
14. *Short Form-36 Health Survey (SF-36)* – Assessment of generic health concepts in adolescents and adults.
15. *TNO-AZL Questionnaire for Adult's Health-Related Quality of Life (TAAQOL)* – Assessment of health status in adults, weighted by the impact of health status problems on well-being.
16. *World Health Organization Quality of Life Assessment Instrument (WHOQOL-100 and WHOQOL-BREF)* – Assessment of individuals' perceptions of quality of life.
