# Supplementary material for: Do sugar-sweetened beverages cause adverse health outcomes in adults? A systematic review protocol
Source: Syst Rev. 2014 Sep 23;3:108. doi: 10.1186/2046-4053-3-108 (PMC4178316; doi:10.1186/2046-4053-3-108)
Supplement: Additional file 4 — Risk of Bias Assessment. This file provides the tools and domains for risk of bias assessment. [file 2046-4053-3-108-S4.pdf]

Additional file 4

<u>The Cochrane Risk of Bias Tool (for RCTs)</u>

SELECTION BIAS

Was a randomly generated sequence used?

Criteria for a judgement of 'Low risk' of bias.

- The investigators describe a random component in the sequence generation process such as: Referring to a random number table; Using a computer random number generator; Coin tossing; Shuffling cards or envelopes; Throwing dice; Drawing of lots; Minimization†.

†Minimization may be implemented without a random element, and this is considered to be equivalent to being random.

Note: If a non-random component in the sequence generation process is indicated, such as a sequence generated by odd or even date of birth, a sequence generated by some rule based on date (or day) of admission, or a sequence generated by some rule based on hospital or clinic record number, then we will designate such studies as non-randomized trials and use the quality checklist provided above to assess risk of bias.

Other non-random approaches happen much less frequently than the systematic approaches mentioned above and tend to be obvious. They usually involve judgement or some method of non-random categorization of participants, for example: Allocation by judgement of the clinician; Allocation by preference of the participant; Allocation based on the results of a laboratory test or a series of tests; Allocation by availability of the intervention.

Criteria for the judgement of 'Unclear risk' of bias.

Insufficient information about the sequence generation process to permit judgement of 'Low

risk’ or ‘High risk’.

Was allocation of the sequence adequately concealed?

Criteria for a judgement of ‘Low risk’ of bias.

Participants and investigators enrolling participants could not foresee assignment because one of the following, or an equivalent method, was used to conceal allocation: Central allocation (including telephone, web-based and pharmacy-controlled randomization); Sequentially numbered drug containers of identical appearance; Sequentially numbered, opaque, sealed envelopes.

Criteria for the judgement of ‘High risk’ of bias.

Participants or investigators enrolling participants could possibly foresee assignments and thus introduce selection bias, such as allocation based on: Using an open random allocation schedule (e.g. a list of random numbers); Assignment envelopes were used without appropriate safeguards (e.g. if envelopes were unsealed or non-opaque or not sequentially numbered); Alternation or rotation; Date of birth; Case record number; Any other explicitly unconcealed procedure.

Criteria for the judgement of ‘Unclear risk’ of bias.

Insufficient information to permit judgement of ‘Low risk’ or ‘High risk’. This is usually the case if the method of concealment is not described or not described in sufficient detail to allow a definite judgement – for example if the use of assignment envelopes is described, but it remains unclear whether envelopes were sequentially numbered, opaque and sealed.

PERFORMANCE BIAS

Were participants and personnel blinded?

Criteria for a judgement of 'Low risk' of bias.

Any one of the following: No blinding or incomplete blinding, but the review authors judge that the outcome is not likely to be influenced by lack of blinding; Blinding of participants and key study personnel ensured, and unlikely that the blinding could have been broken.

Criteria for the judgement of 'High risk' of bias.

Any one of the following: No blinding or incomplete blinding, and the outcome is likely to be influenced by lack of blinding; Blinding of key study participants and personnel attempted, but likely that the blinding could have been broken, and the outcome is likely to be influenced by lack of blinding.

Criteria for the judgement of 'Unclear risk' of bias.

Any one of the following: Insufficient information to permit judgement of 'Low risk' or 'High risk'; The study did not address this outcome.

***Was exposure or compliance measured?**

Was the amount of exposure and, if relevant, subject/patient compliance measured? Was exposure information collected with standard, valid, and reliable measures and conducted consistently across all groups?

Criteria for the judgement of 'Low risk' of bias

Example: compliance with randomized interventions measured and with standard, valid, and reliable measures consistently across groups.

Criteria for the judgement of 'High risk' of bias

Examples: fidelity of the intervention not measured; measures were not standard, valid, and reliable.

Criteria for the judgement of 'Unclear risk' of bias

Insufficient information to permit judgement of 'Low risk' or 'High risk'.

DETECTION BIAS

Were outcome assessors blinded?

Criteria for a judgement of 'Low risk' of bias.

Any one of the following: No blinding of outcome assessment, but the review authors judge that the outcome measurement is not likely to be influenced by lack of blinding; Blinding of outcome assessment ensured, and unlikely that the blinding could have been broken.

Criteria for the judgement of 'High risk' of bias.

Any one of the following: No blinding of outcome assessment, and the outcome measurement is likely to be influenced by lack of blinding; Blinding of outcome assessment, but likely that the blinding could have been broken, and the outcome measurement is likely to be influenced by lack of blinding.

Criteria for the judgement of 'Unclear risk' of bias.

Any one of the following: Insufficient information to permit judgement of 'Low risk' or 'High risk'; The study did not address this outcome.

ATTRITION BIAS

Were incomplete outcome data adequately addressed?

Criteria for a judgement of 'Low risk' of bias.

Any one of the following: No missing outcome data; Reasons for missing outcome data unlikely to be related to true outcome (for survival data, censoring unlikely to be introducing bias); Missing outcome data balanced in numbers across intervention groups, with similar reasons for

missing data across groups; For dichotomous outcome data, the proportion of missing outcomes compared with observed event risk not enough to have a clinically relevant impact on the intervention effect estimate; For continuous outcome data, plausible effect size (difference in means or standardized difference in means) among missing outcomes not enough to have a clinically relevant impact on observed effect size; Missing data have been imputed using appropriate methods.

Criteria for the judgement of 'High risk' of bias.

Any one of the following: Reason for missing outcome data likely to be related to true outcome, with either imbalance in numbers or reasons for missing data across intervention groups; For dichotomous outcome data, the proportion of missing outcomes compared with observed event risk enough to induce clinically relevant bias in intervention effect estimate; For continuous outcome data, plausible effect size (difference in means or standardized difference in means) among missing outcomes enough to induce clinically relevant bias in observed effect size; 'As-treated' analysis done with substantial departure of the intervention received from that assigned at randomization; Potentially inappropriate application of simple imputation.

Criteria for the judgement of 'Unclear risk' of bias.

Any one of the following: Insufficient reporting of attrition/exclusions to permit judgement of 'Low risk' or 'High risk' (e.g. number randomized not stated, no reasons for missing data provided); The study did not address this outcome.

REPORTING BIAS

Is the study free of selective outcome reporting?

Criteria for a judgement of 'Low risk' of bias.

Examples: The study protocol is available and all of the study’s pre-specified (primary and secondary) outcomes that are of interest in the review have been reported in the pre-specified way; The study protocol is not available but it is clear that the published reports include all expected outcomes, including those that were pre-specified (convincing text of this nature may be uncommon).

Criteria for the judgement of ‘High risk’ of bias.

Examples: Not all of the study’s pre-specified outcomes have been reported; One or more outcomes is reported using measurements, analysis methods or subsets of the data (e.g. subscales) that were not pre-specified; One or more reported outcomes were not pre-specified (unless clear justification for their reporting is provided, such as an unexpected adverse effect); One or more outcomes of interest in the review are reported incompletely so that they cannot be entered in a meta-analysis; The study report fails to include results for a key outcome that would be expected to have been reported for such a study. For RCTs with baseline outcome measurements, score high risk if important differences were present but not adjusted for in the analysis.

Criteria for the judgement of ‘Unclear risk’ of bias.

Insufficient information to permit judgement of ‘Low risk’ or ‘High risk’. It is likely that the majority of studies will fall into this category.

***If relevant, when data are analyzed using inferential statistics, was the statistical test appropriate?**

Score ‘Low risk’ of bias if the test was appropriate. Score ‘High risk’ of bias if the test was not appropriate. Score ‘Unclear risk’ if insufficient information provided to make a judgement.

OTHER BIAS

Is the study free of other bias?

Criteria for a judgement of 'Low risk' of bias.

The study appears to be free of other sources of bias.

Criteria for the judgement of 'High risk' of bias.

There is at least one important risk of bias. For example, the study: Had a potential source of bias related to the specific study design used; or has been claimed to have been fraudulent; or had some other problem; or there is a possibility of recruitment bias in a cluster RCT (i.e. participants were recruited into the trial *after* clusters were randomized into intervention or control groups).

Criteria for the judgement of 'Unclear risk' of bias.

There may be a risk of bias, but there is either: Insufficient information to assess whether an important risk of bias exists; or Insufficient rationale or evidence that an identified problem will introduce bias.

* Question not part of the original Cochrane risk of bias tool.

<u>Modified EAL Quality Criteria Checklist (Observational Studies and CCTs)</u>

SELECTION BIAS

1. Was the selection of study subjects/patients free from bias?

- Were inclusion/exclusion criteria specified (e.g., risk, point in disease progression/inception cohort, diagnostic or prognosis criteria), and with sufficient detail and without omitting criteria critical to the study?
- Were criteria applied equally to all study groups? Note: for case-control studies, cases and controls will differ on outcome status.

An example of low risk of bias is when inclusion/exclusion criteria were specified in sufficient details and applied equally to all study groups. An example of high risk of bias is if inclusion/exclusion criteria were not applied equally to all study groups. An unclear risk of bias may be awarded when the information presented does not permit judgement of 'low risk' or 'high risk'.

CONFOUNDING BIAS

1. Were study groups comparable?

- Were distribution of disease status, prognostic factors, and other factors (e.g., demographics) similar across study groups at baseline?
- Were concurrent controls used? (Concurrent preferred over historical controls.)
- If a cohort study, were groups comparable on important confounding factors? If case control study, were potential confounding factors comparable for cases and controls?
- Were adequate adjustments made (at the design or analysis stage) for effects of confounding factors that might have affected the outcomes (e.g., multivariate analyses, stratification, matching, propensity scores, instrumental variables, etc.)?

An example of low risk of bias is if study groups were not comparable for confounding factors but those factors were appropriately adjusted for in the analysis. An example of high risk of bias is if no adjustments were made to account for important differences in confounding variables at baseline. An unclear risk of bias may be awarded when the information presented does not permit judgement of 'low risk' or 'high risk'.

To assess adequacy of adjustment for confounding, we will use the minimal sufficient adjustment set of covariates as informed by outcome specific DAGs reported above. Studies may have been biased by both over and under adjustments in their analyses.

PERFORMANCE BIAS

1. Were subjects, clinicians/practitioners, and investigators blinded to treatment group, as appropriate?
2. If co-interventions were provided/administered, were they provided equally and in a similar manner to all study groups?
3. Were any extra or unplanned treatments provided? Were they balanced across groups?

An example of low risk of bias is if no co-interventions or unplanned treatments were provided in either group and participants were compliant with the intervention. An example of high risk of bias is if contamination of the SSB exposure occurred in the control arm. An unclear risk of bias may be awarded when the information presented does not permit judgement of 'low risk' or 'high risk'.

ATTRITION BIAS

1. Was method of handling withdrawals described?

- Were follow up methods described and the same for all groups?
- Was the number, characteristics of withdrawals (i.e., dropouts, lost to follow up, attrition rate) and/or response rate described for each group? (Follow up goal for a strong study is 80%.)
- Were all enrolled subjects/patients (in the original sample) accounted for?

- Were reasons for withdrawals similar across groups?

2. Was “intent to treat” analysis of outcomes done?

An example of low risk of bias would be if a small and similar proportion of participants were lost to follow-up between groups, with similar reasons for attrition. An example of high risk of bias would be if the proportion of missing outcomes enough to bias the effect estimate. An unclear risk of bias may be awarded when the information presented does not permit judgement of ‘low risk’ or ‘high risk’.

MEASUREMENT (DETECTION) BIAS

1. Was blinding used to prevent introduction of bias?

- Were data collectors/outcomes assessors blinded for outcomes assessment? (Objective outcomes would be determined to be at low risk of bias.)
- In cohort study, were measurements of outcomes blinded?
- In case control study, was case definition explicit and case ascertainment not influenced by exposure status?

2. Were outcomes clearly defined and the measurements valid and reliable?

- Was the period of follow-up long enough for outcome(s) to occur?
- Were the observations and measurements based on standard, valid, and reliable data collection instruments/tests/procedures?
- Were the measurements conducted consistently across groups?

3. * In prospective studies, was the outcome (e.g. weight) measured subjectively (e.g. using patient reports) after assessing SSB consumption (measuring outcome before assessing SSB consumption may cause subjects to modify their subsequent response)?

4. * Has the study taken repeated measures of factors that may vary from period to period (e.g. caloric intake, physical activity) and factored the variation in analyses?
5. Was the amount of exposure and, if relevant, subject/patient compliance measured? Was exposure information collected with standard, valid, and reliable measures? Were exposure measurements conducted consistently across all groups (e.g. the risk for differential recall bias in case-control studies)?
6. Were assessments of confounding variables based on standard, valid, and reliable measures? Were measurements conducted consistently across groups irrespective of exposure status?

An example of low risk of bias is if the outcome is objective and the period of follow-up is sufficient for the outcome to occur. An example of high risk of bias is if the outcomes assessors were not blinded to exposure and outcome measurements were not based on a standard, valid, and reliable instrument. An unclear risk of bias may be awarded when the information presented does not permit judgement of 'low risk' or 'high risk'.

REPORTING BIAS

1. * Is the report free from selective outcome reporting (compare outcomes reported in results with protocol when available or outcomes prespecified in methods or assess reviewers' deliberative judgment as to the likelihood of outcome specific reporting bias given the design and objectives of the study)?
2. Were correct/appropriate statistical tests used and assumptions of tests not violated?

An example of low risk of bias is if outcomes and analyses were conducted as pre-specified and correct statistical tests were used. An example of high risk of bias is if authors omit

reporting an outcome they had prespecified. An unclear risk of bias may be awarded when the information presented does not permit judgement of 'low risk' or 'high risk'.

OTHER BIAS

1. * Is bias due to funding or sponsorship unlikely?
2. * If study is a non-randomized cluster trial, were participants recruited into the trial *before* the clusters were designated as intervention or control sites?

Score at low risk of bias if the study appears to be free of other sources of bias. An example of high risk of bias is if the study is funded by a commercial entity with a vested interest in the outcome. An unclear risk of bias may be awarded when the information presented does not permit judgement of 'low risk' or 'high risk'. Other risks of bias may be reported as encountered in studies.

* Question not part of the original EAL Quality Criteria Checklist.

<u>EPOC Tool (for CBA)</u>

SELECTION BIAS

Was the allocation sequence adequately generated?

CBA studies should be scored "High risk".

Was the allocation adequately concealed?

CBA studies should be scored "High risk".

Were baseline outcome measurements similar?

Score "Low risk" if no important differences were present across study groups for patient outcomes.

CONFOUNDING BIAS

Were baseline characteristics similar?

Score “Low risk” if baseline characteristics of the study and control providers are reported and similar. Score “Unclear risk” if it is not clear in the paper (e.g. characteristics are mentioned in text but no data were presented). Score “High risk” if there is no report of characteristics in text or tables or if there are differences between control and intervention providers. Note that in some cases imbalance in patient characteristics may be due to recruitment bias whereby the provider was responsible for recruiting patients into the trial.

PERFORMANCE BIAS

***Were subjects, clinicians/practitioners, and investigators blinded to treatment group?**

Score ‘Low risk’ of bias if any one of the following: No blinding or incomplete blinding, but the review authors judge that the outcome is not likely to be influenced by lack of blinding; Blinding of participants and key study personnel ensured, and unlikely that the blinding could have been broken.

Score ‘High risk’ of bias if any one of the following: No blinding or incomplete blinding, and the outcome is likely to be influenced by lack of blinding; Blinding of key study participants and personnel attempted, but likely that the blinding could have been broken, and the outcome is likely to be influenced by lack of blinding.

Score ‘Unclear risk’ of bias if any one of the following: Insufficient information to permit judgement of ‘Low risk’ or ‘High risk’; The study did not address this outcome.

Was the study adequately protected against contamination?

Score “Low risk” if allocation was by community, institution or practice and it is unlikely that the control group received the intervention. Score “High risk” if it is likely that the control group received the intervention (e.g. if students rather than schools were allocated). Score “Unclear risk” if contamination could have occurred.

***If co-interventions were provided, were they provided equally and in a similar manner to all study groups?**

Score ‘Low risk’ if the answer to the question is ‘yes. Score ‘High risk’ if the answer is ‘no’. Score ‘Unclear risk’ if insufficient information to score ‘Low risk’ or ’High risk’.

***Were any extra or unplanned treatments provided? Were they balanced across groups?**

Score ‘Low risk’ if extra treatments were balanced across groups. Score ‘High risk’ if extra treatments were not balanced across groups. Score ‘Unclear risk’ if insufficient information to score ‘Low risk’ or ’High risk’.

***Was exposure or compliance measured?**

Was the amount of exposure and, if relevant, subject/patient compliance measured? Was exposure information collected with standard, valid, and reliable measures and conducted consistently across all groups?

Score ‘Low risk’ of bias if compliance with interventions measured and with standard, valid, and reliable measures consistently across groups. Score ‘High risk’ of bias if the fidelity of the

intervention was not measured or measures were not standard, valid, and reliable. Score ‘Unclear risk’ of bias if insufficient information to permit judgement of ‘Low risk’ or ‘High risk’.

DETECTION BIAS

Was knowledge of the allocated interventions adequately prevented during the study?

Score “Low risk” if the authors state explicitly that the outcome variable was assessed blindly, or the outcome is objective, e.g. length of hospital stay. Score “High risk” if the outcomes were not assessed blindly. Score “Unclear risk” if not specified in the paper.

ATTRITION BIAS

Were incomplete outcome data adequately addressed?

Score “Low risk” if missing outcome measures were unlikely to bias the results (e.g. the proportion of missing data was similar in the intervention and control groups or the proportion of missing data was less than the effect size i.e. unlikely to overturn the study result). Score “High risk” if missing outcome data was likely to bias the results. Score “Unclear risk” if not specified in the paper (Do not assume 100% follow up unless stated explicitly).

REPORTING BIAS

Was the study free from selective outcome reporting?

Score “Low risk” if there is no evidence that outcomes were selectively reported (e.g. all relevant outcomes in the methods section are reported in the results section). Score “High risk” if some important outcomes are subsequently omitted from the results or when compared with the protocol or other report of the study. Score “Unclear risk” if not specified in the paper.

***If relevant, when data are analyzed using inferential statistics, was the statistical test appropriate?**

Score 'Low risk' of bias if the test was appropriate. Score 'High risk' of bias if the test was not appropriate. Score 'Unclear risk' if insufficient information provided to make a judgement.

OTHER BIAS

Was the study free from other risks of bias?

Score "Low risk" if there is no evidence of other risk of biases. Parameters from the 'Other Bias' section of the Cochrane risk of bias tool may also be relevant here.

* Question not part of the original Cochrane EPOC tool.

<u>EPOC Tool (for ITS)</u>

Note: If the ITS study has ignored secular (trend) changes and performed a simple t-test of the pre versus post intervention periods without further justification, the study should not be included in the review unless reanalysis is possible.

CONFOUNDING BIAS

Was the intervention independent of other changes?

Score "Low risk" if there are compelling arguments that the intervention occurred independently of other changes over time and the outcome was not influenced by other confounding variables/historic events during study period. If Events/variables identified, note

what they are. Score "High risk" if reported that intervention was not independent of other changes in time.

PERFORMANCE BIAS

***Was exposure or compliance measured?**

Was the amount of exposure and, if relevant, subject/patient compliance measured? Was exposure information collected with standard, valid, and reliable measures and conducted consistently across all groups?

Score 'Low risk' of bias if compliance with randomized interventions measured and with standard, valid, and reliable measures consistently across groups. Score 'High risk' of bias if the fidelity of the intervention was not measured or measures were not standard, valid, and reliable. Score 'Unclear risk' of bias if insufficient information to permit judgement of 'Low risk' or 'High risk'.

ATTRITION BIAS

Were incomplete outcome data adequately addressed?

Score "Low risk" if missing outcome measures were unlikely to bias the results (e.g. the proportion of missing data was similar in the pre- and post-intervention periods or the proportion of missing data was less than the effect size i.e. unlikely to overturn the study result). Score "High risk" if missing outcome data was likely to bias the results. Score "Unclear risk" if not specified in the paper (Do not assume 100% follow up unless stated explicitly).

DETECTION

Was the shape of the intervention effect pre-specified?

Score "Low risk" if point of analysis is the point of intervention OR a rational explanation for the shape of intervention effect was given by the author(s). Where appropriate, this should include an explanation if the point of analysis is NOT the point of intervention; Score "High risk" if it is clear that the condition above is not met.

Was the intervention unlikely to affect data collection?

Score "Low risk" if reported that intervention itself was unlikely to affect data collection (for example, sources and methods of data collection were the same before and after the intervention); Score "High risk" if the intervention itself was likely to affect data collection (for example, any change in source or method of data collection reported).

Was knowledge of the allocated interventions adequately prevented during the study?

Score "Low risk" if the authors state explicitly that the outcome variables were assessed blindly, or the outcomes are objective, e.g. length of hospital stay. Score "High risk" if the outcomes were not assessed blindly. Score "Unclear risk" if not specified in the paper.

REPORTING BIAS

Was the study free from selective outcome reporting?

Score "Low risk" if there is no evidence that outcomes were selectively reported (e.g. all relevant outcomes in the methods section are reported in the results section) or analyses selectively conducted. Score "High risk" if some important outcomes are subsequently omitted from the results. Score "Unclear risk" if not specified in the paper.

***If relevant, when data are analyzed using inferential statistics, was the statistical test appropriate?**

Score 'Low risk' of bias if the test was appropriate. Score 'High risk' of bias if the test was not appropriate. Score 'Unclear risk' if insufficient information provided to make a judgement.

OTHER BIAS

Was the study free from other risks of bias?

Score "Low risk" if there is no evidence of other risk of biases. e.g. should consider if seasonality is an issue (i.e. if January to June comprises the pre-intervention period and July to December the post, could the "seasons' have caused a spurious effect). Parameters from the 'Other Bias' section of the Cochrane risk of bias tool may also be relevant here.

* Question not part of the original Cochrane EPOC tool.
