# Supplementary material for: Do sugar-sweetened beverages cause adverse health outcomes in adults? A systematic review protocol
Source: Syst Rev. 2014 Sep 23;3:108. doi: 10.1186/2046-4053-3-108 (PMC4178316; doi:10.1186/2046-4053-3-108)
Supplement: Additional file 5 — Causal directed acyclic graphs (DAG) depicting the postulated causal and biasing pathways between sugar-sweetened beverage consumption and adverse health outcomes [70-72]. This file provides illustrations and accompanying text of the postulated causal and biasing pathways between sugar-sweetened beverage consumption and adverse health outcomes. [file 2046-4053-3-108-S5.pdf]

Additional file 5

Causal DAG for selected outcomes in adults

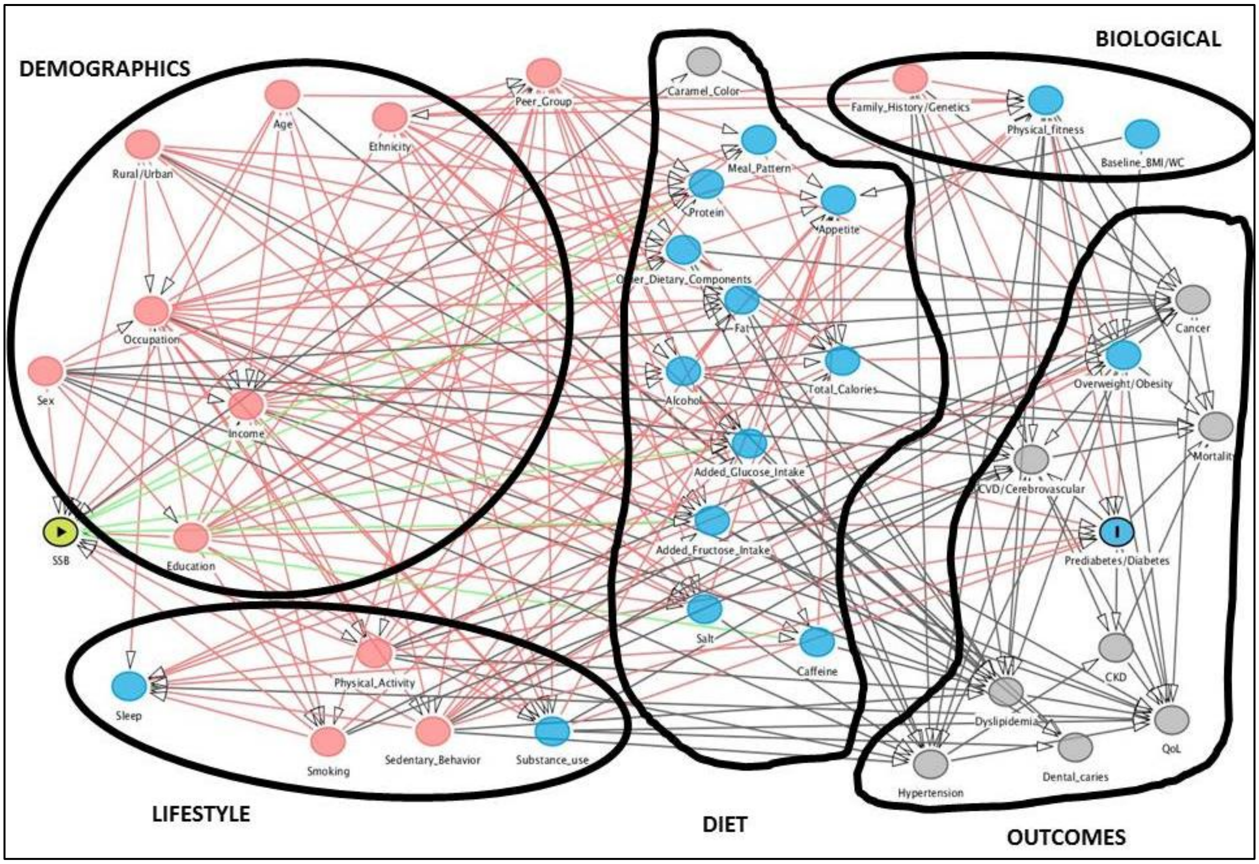

Legend: Exposure Outcome Ancestor of exposure Ancestor of outcome

Ancestor of exposure *and* outcome

Irrelevant variable. Intrinsic to the Dagitty program irrelevant variables appear because they are implicated in a causal or biasing pathway for another outcome in the DAG figure.

Causal path Biasing path

Abbreviations: BMI = body mass index; CKD = chronic kidney disease; CVD = cardiovascular disease; QoL = quality of life; SSB = sugar-sweetened beverage; WC = waist circumference

Minimal sufficient adjustment set for estimating the total effect of SSB consumption on each of the indicated outcomes: Age, Education, Income, Peer Group, Physical Activity, Rural/Urban, Sedentary Behavior, Sex, Smoking

This figure displays the DAG for the effect of SSB consumption on metabolic-related diseases, CKD, cancer, mortality, and quality of life in adults.

Within the demographic variables, income, education, and occupation together describe socioeconomic status. Rural/urban has been included to take into account the effect of the physical environment on disease. Upon reflection, it came to light that there may be a finer distinction to the physical environment than rural/urban, denoted by the neighborhood environment. The neighborhood environment may include factors such as proximity to or density of fast food restaurants and physical recreation facilities. We have currently not included neighborhood environment in the DAG but we will consider doing so on a post-hoc basis if we find that such variables are considered in studies. Since neighborhood environment will likely be assessed at the aggregate level, the issue of ecological fallacy will need to be taken into consideration if it is included post-hoc.

Peer group refers to the behaviours or disease status (e.g. overweight/obese) of subjects' family and friends.

Meal pattern refers to time of day at which and number of meals are consumed.

Within the lifestyle variables, activity level has been divided into physical activity and sedentary behavior. Sedentary behavior is defined as a distinct class of behaviours (for example, watching television or driving) with little physical movement and low energy expenditure (≤ 1.5 metabolic equivalents).[70] Studies have shown a dose-response relationship between sedentary activities and adverse health outcomes.[70] Physical fitness, being a functional measure, is also considered in these pathways but as a biological variable.

Quality of life is a complicated variable that is assessed as a composite of multiple domains, such as mobility, work capacity and satisfaction, social interactions and relationships, performance of daily activities, leisure, self-perception, emotional well-being, and sleep.[71,72] For simplicity, we have omitted displaying these variables and have shown the link between SSB consumption and quality of life as being mediated primarily by the development of the diseases which may be associated with SSB consumption. It is important to keep in mind that, despite the depiction of the direct arrows, disease will likely affect quality of life through one or more of the domains mentioned above. Similarly, although we have depicted direct arrows from income, occupation, and rural/urban to quality of life, their effects will likely be mediated through other factors not shown in the DAG.

Causal DAG for gout in adults

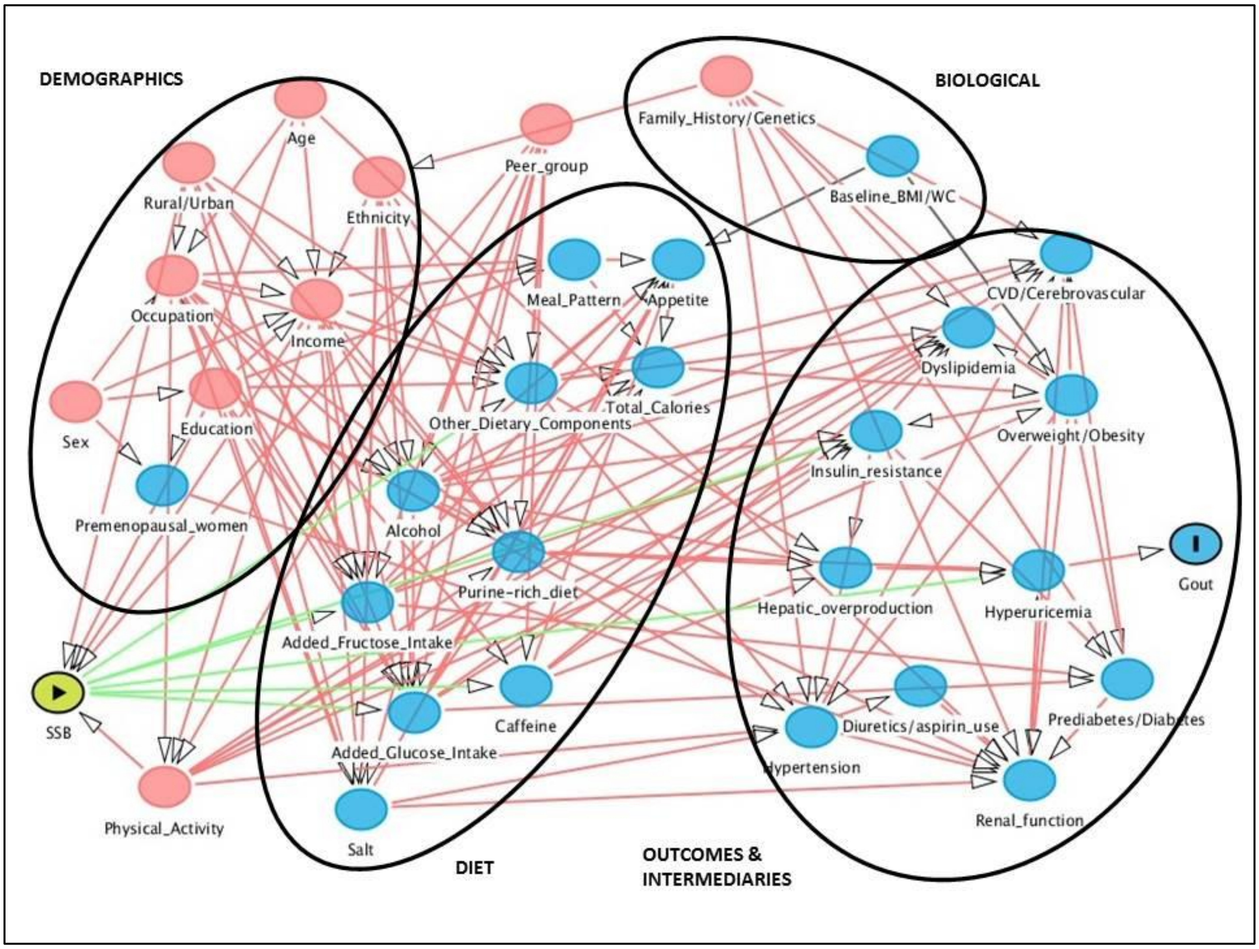

Abbreviations: CVD = cardiovascular disease; SSB = sugar-sweetened beverage

Minimal sufficient adjustment set for estimating the total effect of SSB consumption on gout:

Education, Income, Peer group, Physical activity, Rural/Urban
